# Characteristics of Onefold Clocks of GPS, Galileo, BeiDou and GLONASS Systems

**DOI:** 10.3390/s21072396

**Published:** 2021-03-30

**Authors:** Qingsong Ai, Kamil Maciuk, Paulina Lewinska, Lukasz Borowski

**Affiliations:** 1 State Key Laboratory of Geodesy and Earth’s Dynamics, Innovation Academy for Precision Measurement Science and Technology, Wuhan 430071, China; aiqingsong@asch.whigg.ac.cn; 2 University of Chinese Academy of Sciences, Beijing 100049, China; 3 Department of Integrated Geodesy and Cartography, AGH University of Science and Technology, 30-059 Krakow, Poland; 4 Department of Engineering Surveying and Civil Engineering, AGH University of Science and Technology, 30-059 Krakow, Poland; lewinska.paulina@gmail.com; 5 Department of Computer Science, University of York, York YO10 5DD, UK; 6 Department of Geotechnical Engineering, Faculty of Civil Engineering and Architecture, Lublin University of Technology, 20-618 Lublin, Poland; l.borowski@pollub.pl

**Keywords:** BeiDou, clock, clock analysis, Galileo, GLONASS, GNSS, GPS

## Abstract

This research is focused on searching for frequency and noise characteristics for available GNSS (Global Navigation Satellite Systems). The authors illustrated frequency stability and noise characteristics for a selected set of data from four different GNSS systems. For this purpose, 30-s-interval clock corrections were used for the GPS weeks 1982–2034 (the entirety of 2018). Firstly, phase data (raw clock corrections) were preprocessed for shifts and removal of outliers; GLONASS and GPS satellites characterize a smaller number of outliers than BeiDou and Galileo clock products. Secondly, frequency and Hadamard deviation were calculated. This study concludes that the stability of GPS and Galileo is better than that of BDS (BeiDou Navigation Satellite System) and GLONASS. Regarding noise, the GPS, Galileo, and BDS clocks are affected by the random walk modulation noise (RWFM), flashing frequency modulation noise (FFM), and white frequency modulation noise (WFM), whereas the GLONASS clocks are mainly affected only by WFM.

## 1. Introduction

The IGS (International GNSS Service) has provided satellites’ precise orbits and products since 1994 and 2004, respectively [1]. Along with the development of other satellite systems such as the European Galileo system, the Chinese BeiDou, or the Japanese QZSS (Quasi-Zenith Satellite System), IGS initiated the introduction of the Multi-GNSS EXperiment (MGEX) products. It is worth mentioning that MGEX clock offset products and orbits are provided by several analysis centers [2]. In this paper, the authors present a quality analysis of the precise clock products provided by the MGEX, based on the occurrence of clock offset. The obtained results are used to analyze the stability and noise of GPS, GLONASS, Galileo, and BeiDou satellites.

Onboard atomic clocks play a key role in GNSS positioning. The quality of GNSS performance depends heavily on satellite clocks [3,4]. In recent years, there have been a number of publications containing clock stability analyses, but the majority of them cover only one or two GNSS systems [5,6,7,8]. Wang et al. [9] analyzed BDS clocks with the use of Allan deviation, and similar research was conducted by Steigenberger [10], but using Galileo clocks. Comparisons of the two GNSS clocks were provided, e.g., by Ai [2] (GLONASS and Galileo) or by Daly [11] (GPS and GLONASS). GPS and GLONASS were the first satellite navigation systems, as well as the first to become fully operational. The significant development of the BeiDou and Galileo systems has taken place only in recent years. Thus, available products, receivers, and software for processing the signals of those systems also came later.

This paper contributes to the existing knowledge by analyzing four different GNSS clocks and completing a long-term analysis (1 year) with a very low sampling interval (30 s), which leads to more than 1 M observations per satellite. To the best of the authors’ knowledge, this kind of complex analysis of these four systems has neither been done nor published yet. The adopted methodology includes the Hadamard deviation, the MAD (Median Absolute Deviation) method, and the lag 1 ACF (autocorrelation function) for clock correction data preprocessing for noise identification.

## 2. Materials and Methods

There are currently several dozen GNSS satellites in orbit. GPS and GLONASS satellites carry Rb (rubidium) and Cs (caesium) frequency standards, whereas Galileo satellites are equipped with PHM (passive hydrogen maser) and Rb clocks. In the case of BeiDou, the clocks used are of either Rb or HMAC (Hydrogen Maser Atomic Clock) standards [12]. Typically, after clock trend removal, PHM clocks are up to two orders of magnitude more precise than Rb or Cs clocks [13]. Clock stabilities are typically measured on the basis of phase-to-frequency data conversion using Allan or Hadamard (and related) variances [14,15]. Phase data $x_{i}$ may be converted into frequency data $y_{i}$ by first taking the difference of the phase data and dividing them by the sampling interval τ, giving the formula:

(1) $$y_{i}=\frac{x_{i+1}-x_{i}}{\tau }$$

One of the most crucial indicators of atomic clock performance is frequency stability, which is usually expressed by Allan and Hadamard variances. As for the frequency, drift is easily ignored by the Allan variance, and the Hadamard variance is insensitive to linear frequency drift [16,17]. The Hadamard variance adopts the second difference of the fractional frequencies. Such a property is useful for stability analysis of the rubidium atomic clocks and passive hydrogen masers [18]. The stability of GPS, BDS, and Galileo clocks are all efficient through the use of the HDEV (Hadamard Deviation) $H\sigma_{y}^{2}\left(\tau \right)$; is the square root of HVAR; thus, in order to unify for the evaluating method, such variance is used for the following stability analysis.

(2) $$H\sigma_{y}^{2}\left(\tau \right)=\frac{1}{6\left(M-2\right)}\overset{M-2}{\underset{i=1}{\sum }}{\left[y_{i+2}-2y_{i+1}+y_{i}\right]}^{2}=\frac{1}{6\tau^{2}\left(N-3m\right)}\overset{N-3}{\underset{i=1}{\sum }}{\left[x_{i+3}-3x_{i+2}+3x_{i+1}-x_{i}\right]}^{2}$$

HDEV can be acquired by Equation (2) based on the fractional frequencies $y_{i}\left(m\right)\;$or the clock offset series $x_{i}$, where M is the amount of $y_{i}\left(m\right)$, which is spaced by the measurement interval τ, N=M+1 is the number of clocks offset series $x_{i}$(*m*), and m is the averaging factor.

GFZ (Geoforschungszentrum Potsdam) has supplied Multi-GNSS products since 2014. They have a highly precise clock offset with an RMS (Root-Mean-Square) of less than 0.1 ns and can accurately evaluate atomic clock performance. In this study, we used the Multi-GNSS precise clock offset products by GFZ from 1 January 2018 to 31 December 2018 (GPS weeks 1982–2034), to fully display the performance of GNSS satellite clocks [19]. For this period, 83 GNSS signals were available: 32 GPS, 23 GLONASS, 18 Galileo, and 10 BeiDou. The analysis consisted of two parts: Determination of the magnitude of outliers based on frequency data and testing of clock stability using HDEV (Hadamard deviation), which is the square root of Equation (2).

For each of the analyzed GNSS, 3 representative satellites were randomly selected due to the different number of satellites in orbit in each GNSS, since visualization of data from more than 100 satellites would be difficult. Onboard oscillators are equipped with 3 different frequency standards:

- Rb (Rubidium)—BeiDou satellites C09, C10, C12, and GPS satellites G10, G14, G25;
- Cs (Caesium)—GLONASS R07, R10, R11;
- PHM (Passive hydrogen maser)—Galileo E02, E03, E04.

As for the long-term, on-board operation of the atomic clock, the satellite clock offset series commonly includes clock jump, gross error, and phase discontinuity. To effectively use the clock offset series and accurately obtain performance stability, the outliers in its products need to be detected before analysis. Generally, the MAD [20] method is adopted to detect gross errors. It can be expressed as:

(3) $$MAD=Median\frac{\left|y_{i}-m\right|}{0.6745}$$

where MAD is set as the threshold value for the gross error detection and m is the median of $y_{i}$. Then, the outlier $y_{i}$ is filled by the linear function. Meanwhile, the frequency which causes frequency jump is removed to ensure a credible performance. The following stability and noise analysis are entirely based on the corrected data.

**Figure 1:**
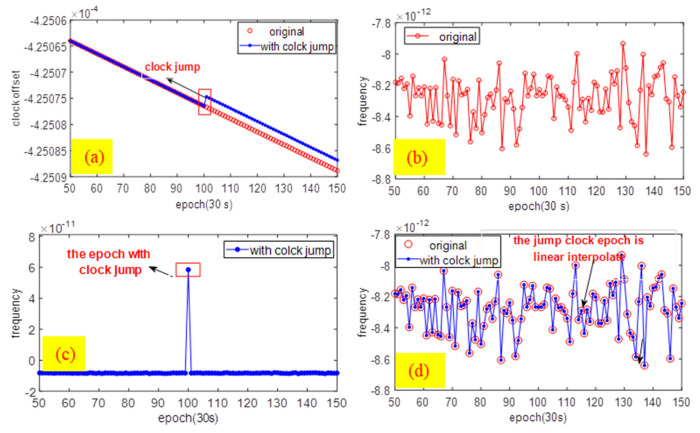
The clock offset and frequency series in the situation of clock jump.

As for the clock jump in Figure 1, the analysis centers adopt single-day observations to estimate the clock offset and the orbit, which leads to a certain bias and gap in the daily clock offset. They can directly influence the continuity of the clock offset series and then affect applications of the satellite clock, such as precise point positioning, so we use clock error prediction. It is worth mentioning that, for stability and noise analysis, if the clock jump is not caused by a frequency jump, the characterization analysis will not be affected. It is preprocessed as a gross error before the examination of the stability and noise analysis. The gross jump is interpolated by a linear function that does not affect performance and noise identification.

As per the red part of Figure 1a and Figure 1b, the original clock and the frequency have normal status (clock jump); the blue line shows a clock jump at the 100th epoch. Consequently, the frequency series (Figure 1c) has an abnormal jump at the 100th epoch, which is obviously inconsistent with other epochs. This epoch was treated as a gross error, after which we preprocessed the clock series by linear interpolation.

As shown in Figure 1d, the frequency sequence with the clock jump was adequately removed. Then, the processed frequency was consistent with the original frequency; thus, the clock jump did not affect the stability and noise identification.

**Figure 2:**
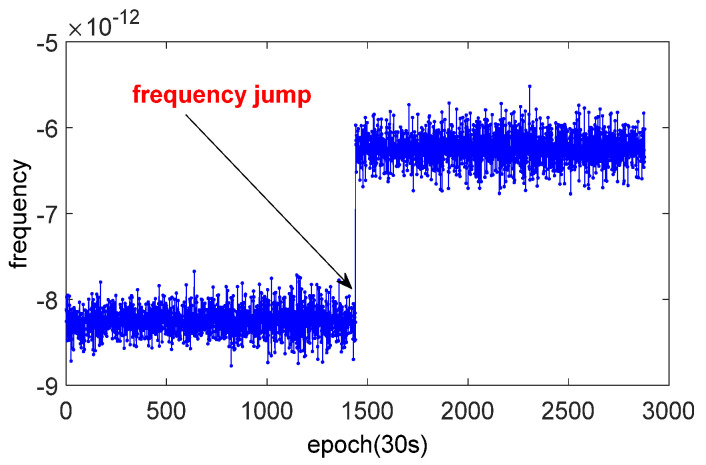
Frequency jump of satellite clock.

However, the frequency jumps in Figure 2, for satellite clocks, occurred for three reasons [21]: (i) the phase jump in the master control station, (ii) operation frequency modulation to maintain frequency accuracy, and (iii) the switch of the on-board clock (the switching of the clock might be reflected in the change of frequency slope). Thus, the analysis of this situation should be divided into two parts.

## 3. Stability of GNSS Satellite Clock Offset

**Figure 3:**
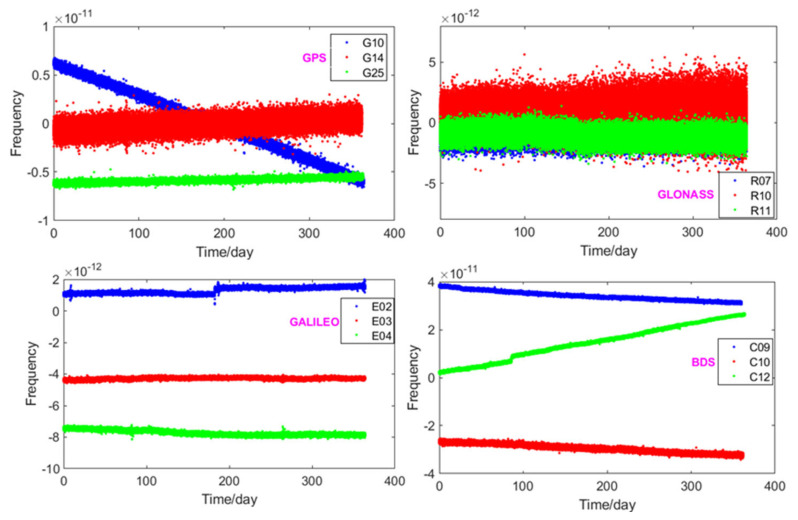
The frequency of GNSS satellites.

Before the stability evaluation, we analyzed the frequency of the GNSS on-board satellite clocks, which is shown in Figure 3. As shown in this figure, the BDS and GPS are equipped with the Rb atomic clocks and the Galileo with the PHM, which have significant frequency drifts (frequency has a certain degree of inclination). The GLONASS are all equipped with the Cs atomic clock—it is free from frequency drift, and the frequency has high consistency between satellites. The BDS frequency has a different bias, close to ± 4 × 10^−11^. The GPS and the Galileo frequencies also have a certain different bias, with a magnitude of 10^−12^, which is not as significant as that of the BDS. The frequency jump of C12 on 27 March 2018 corresponds to the circumstance (ii) of frequency jump, which will greatly affect the stability result. The common method is to divide the frequency into two parts and then obtain the mean of the two-part stability.

**Figure 4:**
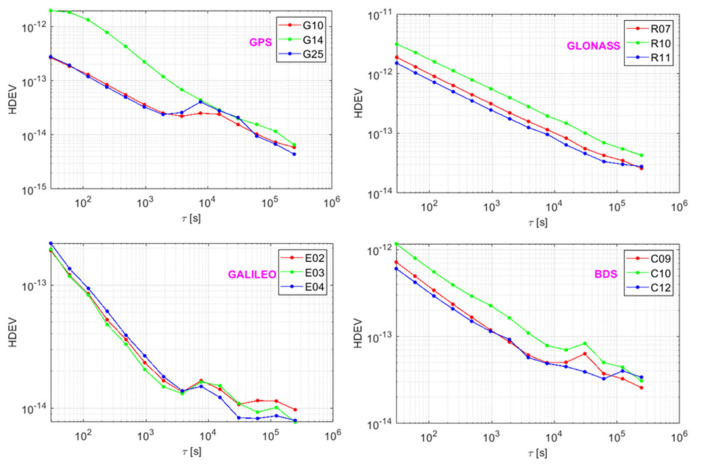
Stability of GNSS satellite clock.

As shown in Figure 4, the stability of the BDS was consistent between satellites; it was approximately 10^−13^ at τ = 100 s and τ = 1000 s, while for 10,000 s and τ, it was one day (τ = 86,400 s), and the result was in the order of 10^−14^. For Galileo, it was 10^−13^ for an average time of τ = 100 s, and 10^−14^ for τ = 1000 s and τ = 10,000 s, respectively, and the day stability was at the magnitude of 10^−15^. The GPS clock G10 and G25 (IIF Rb clock) had a magnitude superior to that of the G14 (IIR Rb clock) with the τ = 30 s to τ = 1000 s; thus, the IIF Rb clock was more stable than the IIR Rb clock. On the other hand, the G14 has a long orbit operation of nearly 20 years [12,22]. The GPS stability of τ=10,000 s was in the order of 10^−14^, and it was around 10^−15^ for τ = 1 day. With the increased averaging time, the number of sampling nodes decreased, and there was a small fluctuation in frequency ${\bar{y}}_{i+m}$ that led to the fluctuation of stability. Similar to the trend of the GPS, the Galileo and BDS slowed down, and the stability curves were non-monotonic from the average time of τ = 10,000 s.

The GLONASS’s stability revealed an exponential linear change with the increase in the averaging time and each clock showed good consistency. This was due to the fact that the GLONASS are equipped with the same type of Cs atomic clock. The GLONASS’s stability was maintained at 10^−12^–10^−13^ for τ = 100 s, while for τ = 1000 s and one day, it was at 10^−13^ and 10^−14^, respectively. From the results, we find that the short-term (τ = 100 s) and medium–long-term (τ = 10,000 s) stability of GPS, Galileo, and BDS were at the level of 10^−13^ and 10^−14^, which was nearly one magnitude of superiority in performance compared to that of the GLONASS. The day stability of BDS and GLONASS was maintained at 10^−14^; thus, it was worse than 10^−15^ for GPS and Galileo. Overall, the stability of GPS and Galileo was better than that of BDS and GLONASS.

## 4. Noise Determination

In the research on noise, many studies have focused on the Allan and modified Allan variance [23,24]. As shown in Figure 4, this method is used to fit in line with the result of the variances and the averaging time, and then to determine the type of noise based on the fitted line. This method is based on the manual result and thus might not reflect the noise characteristic at each averaging time and could be easily affected by the stability result. Howe et al. [16] adopted the B1 (Bias 1: $B1\left(N,r=1,\mu \right)=\frac{N\left(1-N^{\mu }\right)}{2\left(N-1\right)\left(1-2^{\mu }\right)}$) partial factor for noise analysis, which was unable to distinguish the flicker frequency modulation noise and white phase modulation noise, while the averaging time was equal to that of the sample; the frequency drift should have been eliminated before the analysis. The type and magnitude of the abnormal noise with clock aging and environment change directly influences the stability of the atomic clock. In this paper, the lag 1 autocorrelation algorithm is introduced. Unlike the Allan variance method, it can directly obtain the noise type in a consistent and analytic manner. It often modes the dominant power law noise process (RWFM (frequency random walk modulation noise), FFM (flashing frequency modulation noise), WFM (white frequency modulation noise), FPM (flashing phase modulation noise), and WPM (white phase modulation noise)) of the spectral density of the fractional frequency fluctuations, $S_{y}\left(f\right)=\overset{2}{\underset{\alpha =-2}{\sum }}h_{\alpha }f^{\alpha }=h_{-2}f^{-2}+h_{-1}f^{-1}+h_{0}f^{0}+h_{1}f^{1}+h_{2}f^{2}$ to perform a noise analysis. Table 1 lists the relationship of power, lower index α, and noise type.

The power law spectral density $S_{y}\left(f\right)$ is of a single frequency with the unit 1/Hz, the Fourier frequency $f^{\alpha }$ has the unit of Hz, the integral coefficient α is the index of noise type, and the $h_{\alpha }$ is the coefficient of noise intensity.

The second-order stationary condition can be mistaken for the noise of G10 and E02; when the averaging time is around 2 × 10^4^ s, the index of noise type α shows a local maximum. This is owing to the satisfaction of the stationary status σ<0.25; the time series $z_{k}$ should make a difference between epochs, so then the value of d changes to d + 1, which will cause the local jump of p for the formula p=−round(2σ)−2d.

As for the lag 1 autocorrelation law noise identification algorithm, the autocorrelation function (ACF) is presented as [2]:

(4) $$\rho =\frac{E\left[\left(z_{k}-\mu \right)\left(z_{k+1}-\mu \right)\right]}{\sigma_{z}^{2}}$$

where the independent variable $z_{k}=\frac{1}{m}\overset{m}{\underset{i=1}{\sum }}X_{\left(k-1\right)m+i}\left(k=1,2,\ldots ,n\right)$ and μ is separated, the mean value of the time series $X_{k}$ with the average factor m and the mean $z_{k},$ E, and $\sigma_{z}^{2}$ are, respectively, the expectation and variance of $z_{k}$, and $\bar{z}\;$is the mean of $z_{k}$. The autocorrelation function can also be expressed by $r_{1}$, as follows:

(5) $$r_{1}=\frac{\frac{1}{N}\sum_{k=1}^{N-1}\left(z_{k}-\mu \right)\left(z_{k+1}-\mu \right)}{\frac{1}{N}\sum_{k=1}^{N}{\left(z_{k}-\bar{z}\right)}^{2}}$$

where N is the number of points with the average factor m. In the lag1 ACF, the symbol z is a function of autocorrelation value $r_{1}$ with the form of $\sigma =\frac{r_{1}}{1+r_{1}}$; symbol σ is not the standard deviation but a flag for stationary status. The meaning of symbols m and d is shown in the flowchart of the lag 1 ACF Algorithm 1. The power law noise is identified by the form lag1 ACF $r_{1}=\frac{\sigma }{1-\sigma }$ with the stationary criterion of σ<0.5, and the stationary status can be obtained by the form of $\sigma =\frac{r_{1}}{1+r_{1}}$. However, for the divergent situation (σ≥0.5), the first difference is made for the stationary status σ<0.25. The noise judgement criterion is p=−round(2σ)−2d, and the round(2σ) means to obtain the nearest integer, whereas d is the differencing order to obtain the stationary status. The power law index α result is equal to *p* + 2 or *p* for phase or frequency data, respectively, and may be rounded to an integer (although the fractional part is useful for estimating mixed noises) [20]. A detailed flowchart is shown in Algorithm 1.
**Algorithm 1.** Flowchart of lag1 ACF algorithm for noise identification.Done=False, d=0While Not Done$Z_{i}=\frac{1}{M}\overset{M}{\underset{j=1}{\sum }}X_{\left(i-1\right)\cdot M+j}$  (i=1,2,3,…,N)$\bar{Z}=\frac{1}{N}\overset{N}{\underset{i=1}{\sum }}Z_{i}$       $N=int\left(\frac{N_{X}}{M}\right)$$r_{1}=\frac{\sum_{k=1}^{N-1}\left(Z_{k}-\bar{Z}\right)\left(Z_{k+1}-\bar{Z}\right)}{\sum_{k=1}^{N}{\left(Z_{k}-\bar{Z}\right)}^{2}}$  $\sigma =\frac{r_{1}}{1+r_{1}}$$If\;d\geq d_{min}\;\&\left(\sigma <0.25|d\geq d_{max}\right)$
  p=round(2σ)−2d
  Done=TrueElse
  $Z_{1}=Z_{2}-Z_{1,}\ldots ,Z_{N-1}=Z_{N}-Z_{N-1}$
  N=N−1, d=d+1End ifEnd While

**Figure 5:**
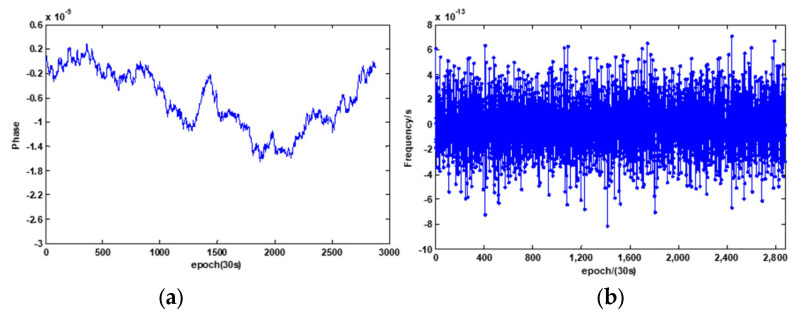
(**a**) Simulated phase and (**b**) frequency series.

To validate the feasibility of the lag1 ACF method, a simulation of a phase data with a noise level of 3 × 10^−12^ τ ^−1/2^ (averaging time is 30 s) is shown in Figure 5a, which corresponds to the frequency in Figure 5b.

**Figure 6:**
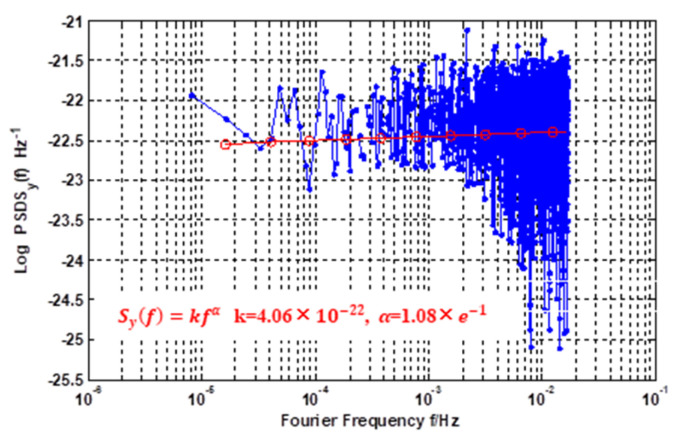
Power spectral density result of simulated frequency.

The analysis of the PSD (power spectrum density) of the simulated data is presented in Figure 6. As shown in Figure 6, the slope of the PSD (α = 0.108) is categorized as WFM noise (α = 0), as in Table 1.

**Figure 7:**
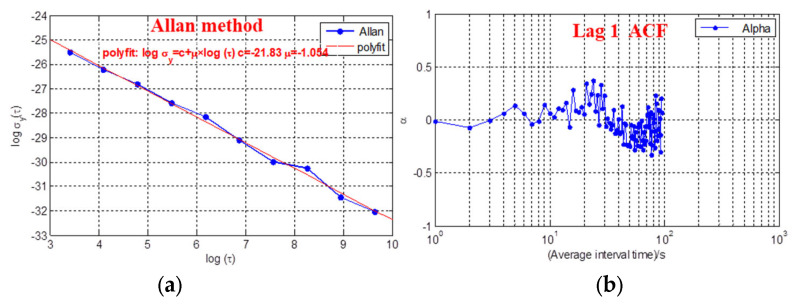
(**a**)Power spectral density and (**b**) lag 1 method noise result of simulated frequency.

The fitting value of the classical Allan variance method can be found in Figure 7a, which is derived from WFM noise (μ=−1.054, around −1). Thus, this method can roughly determine the noise type. Additionally, as in Figure 7b, the results of the lag 1 ACF methods (α is range of ±0.5, around to 0) are generally consistent with those of the PSD algorithm. However, the method of lag 1 ACF can obtain the noise information for each averaging time automatically.

**Figure 8:**
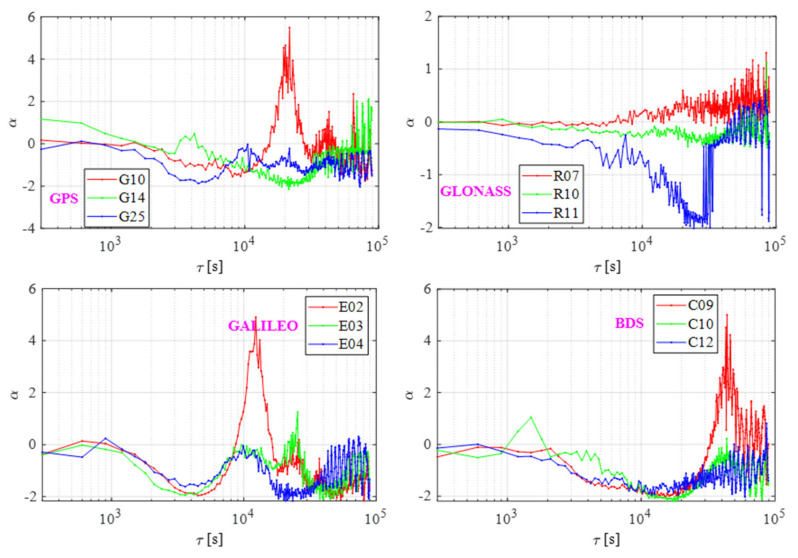
Noise of GNSS satellites.

As for the result in Figure 8, the BDS clocks were mainly affected by WFM when the average time was less than 3 × 10^3^ s. When the averaging time increased, the noise type changed to either FFM or FPM, and C09 and C10, with relatively large frequency, underwent local severe fluctuation after an averaging time of more than 2 × 10^4^ s.

It is owing to the lag 1 ACF method—which limits the condition—that the number of sampling points N must be more than 32 to guarantee freedom [20]. In general, BDS satellite clocks are commonly affected by the combined effect of RWFM, FFM, and WFM. The noise of GPS and Galileo were almost the same, with BDS affected by the three kinds of FM noise, but the noise of Galileo had no local maximum, which may be related to the indistinctive frequency. In the case of GLONASS, the noise types were stable but, when the average time was increased, they became affected by the WFM.

## 5. Conclusions

The MGEX products of all currently available GNSS clocks were analyzed in this paper. Due to the different number of active satellites for each system, three random satellites were selected, and the performance of these satellites is presented in this article. As for the short-term and medium–long-term stability of GPS, Galileo and BDS were at the level of 10^−13^ and 10^−14^, respectively, which had one order of superiority over that of GLONASS. The day stability of BDS and GLONASS was at the same magnitude of 10^−14^, which was worse than the level 10^−1^^5^ of GPS and Galileo. Overall, the stability of GPS and Galileo was better than that of BDS and GLONASS at each averaging time. The GLONASS satellites are all equipped with Cs atomic clocks, which are free from frequency drift, so, the frequency has high consistency between satellites, and the stability is significantly improved, with the increase in the averaging time. The frequency of GLONASS and of Galileo had a certain difference, at a magnitude of 10^−12^, which was not as significant as the BDS and GPS, with a magnitude of 10^−11^. The noise of these two frequency-indistinctive systems was relatively consistent and had no severe fluctuation. The GPS and BDS clocks were mainly influenced by the RWFM, FFM, and WFM noise, and the noise had a certain fluctuation with an average time of more than 2 *×* 10^4^ s. Moreover, the GLONASS noise was stable and only affected by the WFM. Therefore, there may be some correlation between the noise variation and the frequency scale.

## Figures and Tables

**Table 1:** Clock noise types, their Allan deviation slopes, and power spectral density coefficient indices.

μ (Allan Method)	−2	−2	−1	0	1
α (lag 1)	2	1	0	−1	−2
Noise Type	WPM	FPM	WFM	FFM	RWFM

## Data Availability

Not applicable.
